# Magnetic seed versus skin tattoo localization of non-palpable breast lesions: a single institution cohort study

**DOI:** 10.1007/s00330-023-10008-4

**Published:** 2023-08-01

**Authors:** Anna D’Angelo, Lorenzo Scardina, Simone Palma, Stefano Lo Cicero, Alessandro Maresca, Flavia Caprini, Ersilia Biondi, Antonio Franco, Daniela Terribile, Gianluca Franceschini, Paolo Belli, Riccardo Manfredi

**Affiliations:** 1 Dipartimento Di Diagnostica Per Immagini, Fondazione Policlinico Universitario “A. Gemelli” IRCCS, Radioterapia Oncologica Ed Ematologia, Rome, Italy; 2 Division of Breast Surgery, Department of Woman and Child Health and Public Health, Fondazione Policlinico Universitario “A. Gemelli” IRCCS, Rome, Italy

**Keywords:** Breast cancer, Magnetics, Tattooing, Breast-conserving surgery, Mastectomies

## Abstract

**Objective:**

The objective of this retrospective study was to investigate the accuracy and feasibility of magnetic seed compared to skin tattoo in preoperative localization of impalpable breast lesions in terms of accuracy of placement, re-excision and positive margins rates, and breast/surgical specimen volume ratio.

**Methods:**

We retrospectively analyzed 77 patients who underwent breast conservative surgery in our center from November 2020 to November 2021, with previous localization with skin tattoo or magnetic seed.

**Results:**

Thirty-seven magnetic seeds were placed in 36 patients (48.6%) and 40 skin tattoos were performed in the remaining cases (51.4%). The seeds were placed correctly at the two-view mammogram acquired after the insertion in 97.6% (36/37) of cases. With both methods, 100% of the index lesions were completely removed and found in the surgical specimen. The reported re-excision rate was 0% for both groups. A significant difference was observed in the volume of breast parenchyma removed between the two groups, inferior in the seed group (*p* = 0.046), especially in case of voluminous breasts (*p* = 0.003) and small lesions (dimension < 8 mm, *p* = 0.019).

**Conclusions:**

Magnetic seed is a non-radioactive localization technique, feasible to place, recommended in case of non-palpable breast lesions, saving the breast parenchyma removed compared with skin tattoo, without reducing the accuracy.

**Clinical relevance statement:**

Our findings contribute to the current evidence on preoperative localization techniques for non-palpable breast lesions, highlighting the efficacy of magnetic seed localization for deep and small lesions.

**Key Points:**

*• Magnetic seed is a non-radioactive technique for the preoperative localization of non-palpable breast lesions studied in comparison with skin tattoo.*

*• Magnetic seed is feasible to place in terms of post-placement migration and distance from the target lesion.*

*• Magnetic seed is recommended in case of non-palpable breast lesions, saving the breast parenchyma removed without reducing the accuracy.*

**Supplementary information:**

The online version contains supplementary material available at 10.1007/s00330-023-10008-4.

## Introduction

The constant improvement of imaging techniques and the introduction of mammography screening have led to an increased number of diagnosis of non-palpable breast cancers. Currently, about 30% of all breast cancers are not palpable at the time of diagnosis [1]. Non-palpable lesions cause an increased risk of positive tumor margins or excision of healthy parenchyma [2], resulting in high risk of local recurrence, poor cosmetic outcome, and patient dissatisfaction [3]. Adequate preoperative tumor localization is mandatory to perform an appropriate conservative surgery, avoiding loco-regional recurrence and poor esthetic outcomes. Nowadays, there are different techniques of localization: wire localization, carbon marking, radio-guided occult localization (ROLL), radioactive seed localization (RSL), non-radioactive radar localization, magnetic seed localization, intraoperative ultrasound, preoperative skin tattoo, and so on. The choice of which one to use depends on surgeon’s and radiologist’s experience, skills, and available technologies. The most used method is the wire-guided localization (WGL) [4, 5], despite the several disadvantages (e.g., patient discomfort and workflow scheduling) [6–8].

In our center, preoperative localization with skin tattoo, although it is a little-used technique, is the preferred method by surgeon and radiologist for the excision of non-palpable breast lesions, due to the low cost and their skills. In the last years, the magnetic seed (Magseed®, Endomagnetics) has been introduced in our center. In 2016, the Food and Drug Administration (FDA) approved the first magnetic seed marker with the Sentimag® localization probe [3, 9] which has gained considerable interest. The magnetic seed is a 5 × 1 mm stainless steel paramagnetic seed delivered in a sterile 18-G introducer needle [9]. The seed has no intrinsic magnetic activity. When inserted under ultrasound or stereotaxic guidance for the localization of non-palpable breast lesions, the seed is detected with a magnetic detection probe (Sentimag®) during surgery.

The aim of this retrospective single-center study was to evaluate the clinical safety and utility of the magnetic seed location system compared to skin tattoo in localization of non-palpable breast lesions in terms of accuracy of initial placement, re-excision rate, negative margin, and breast/surgical specimen volume ratio.

## Materials and methods

### Patients

In this retrospective single-center study, we included consecutive patients with non-palpable breast lesions who underwent surgery in our center between November 2020 and November 2021. This study was conducted according to the guidelines of the Declaration of Helsinki and approved by the Institutional Review Board. Written informed consent was obtained from all patients.

The preoperative histological diagnoses were breast cancers or B3 lesions [10].

Inclusion criteria were as follows: age 18 years old or older, non-palpable lesions, preoperative localization carried out with skin tattoo or magnetic seed, and surgery performed in our center. Exclusion criteria were the following: palpable lesions and previous chemotherapy treatment.

Skin tattoo and Magseed are the localization techniques used in our center for the preoperative localization of non-palpable breast lesions. The multidisciplinary team (MDT) with radiologists, pathologists, and breast surgeons has defined the localization technique choosing the most precise methods to localize the lesion according to breast size, depth of the lesion from the skin surface, lesion detectability, surgical skills, and available technologies. Specifically, the seed is preferred in breast lesions surgically more difficult to detect, particularly in case of smaller and deeper lesions (not greater than 40 mm depth), in medium–large breasts, in accordance with the surgeon skills. Data were retrospectively collected on patient demographics, cancer characteristics, and surgical details. The time of seed and skin tattoo placement, the time between localization procedure and surgery, and the surgical time were evaluated.

The volume of the mammary gland was calculated considering it as a cone ($\frac{1}{3}$ $\uppi$ ϒ^2^h) from the mediolateral oblique view of staging mammogram [11]. The intraoperative widening and re-excision rate were reported.

Concerning the breast sizes, there was a high percentage of small breasts (A-cup, 47.5%) in the skin tattoo group and medium breasts (B-cup, 62.2%) in the seed group (Table S1). The depth of the lesions from the skin surface was different between the two groups, with lesions deeper in the Magseed group (mean depth was 15.7 mm and 12.2 mm respectively for the seed and skin tattoo group) (Table S1). Moreover, magnetic seed was placed more frequently in patients with small lesions (smaller than 5 mm in 18.9% of cases; Table S2).

### Magseed localization

**Fig. 1:**
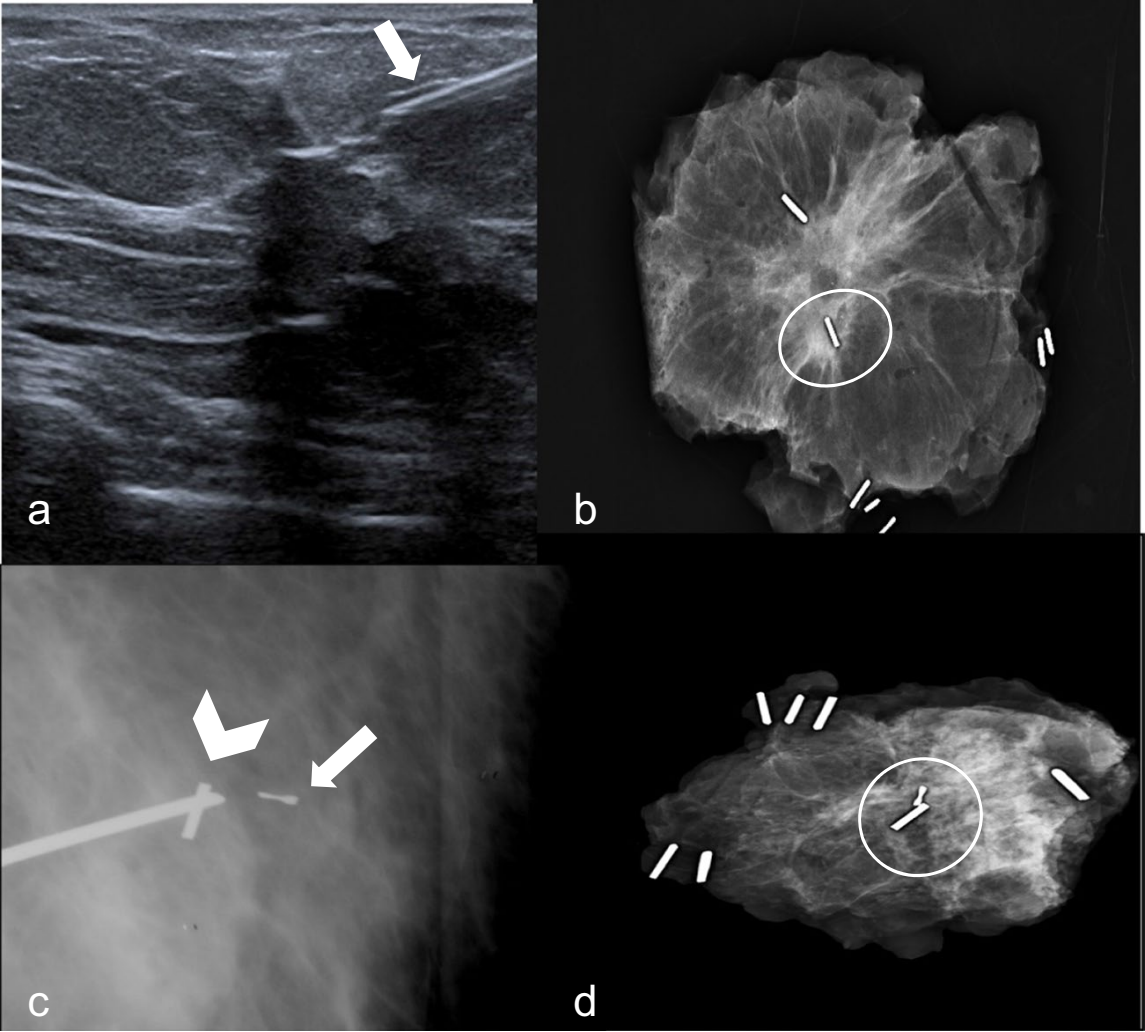
Magseed placement. Ultrasound guidance Magseed placement in the lower inner quadrant of the right breast (**a**, arrow shows the needle). Specimen radiography with the lesion and Magseed correctly excised (**b**, circle shows the seed). Stereotactic guided Magseed placement (**c**, arrowhead) in a 46-year-old woman with breast architectural distortion (radial scar) with the clip (**c**, arrow) placed in the biopsy site. Clip, Magseed, and lesion were found in the specimen radiogram (**d**, circle)

Magnetic seed was placed after disinfection of the skin (chlorhexidine) and the injection of local anesthesia (Mepicain 2%), under ultrasound or stereotactic guidance depending on the MDT decision (Fig. 1). The seed can be deployed by an 18-G preloaded needle of different lengths according to different breast sizes. For lesions with significant size or widespread microcalcifications, bracketing may be performed placing multiple seeds at a close distance ≤ 2 cm to avoid the signal overlap from the different markers [12]. The feasibility of the placement was evaluated following magnetic seed insertion, with mammogram in double projection (mediolateral oblique and cranio-caudal views) acquired to confirm correct placement of the seed, and to exclude marker migration. The position of the Magseed was considered correct if ≤ 10 mm from the lesion.

### Skin tattoo

**Fig. 2:**
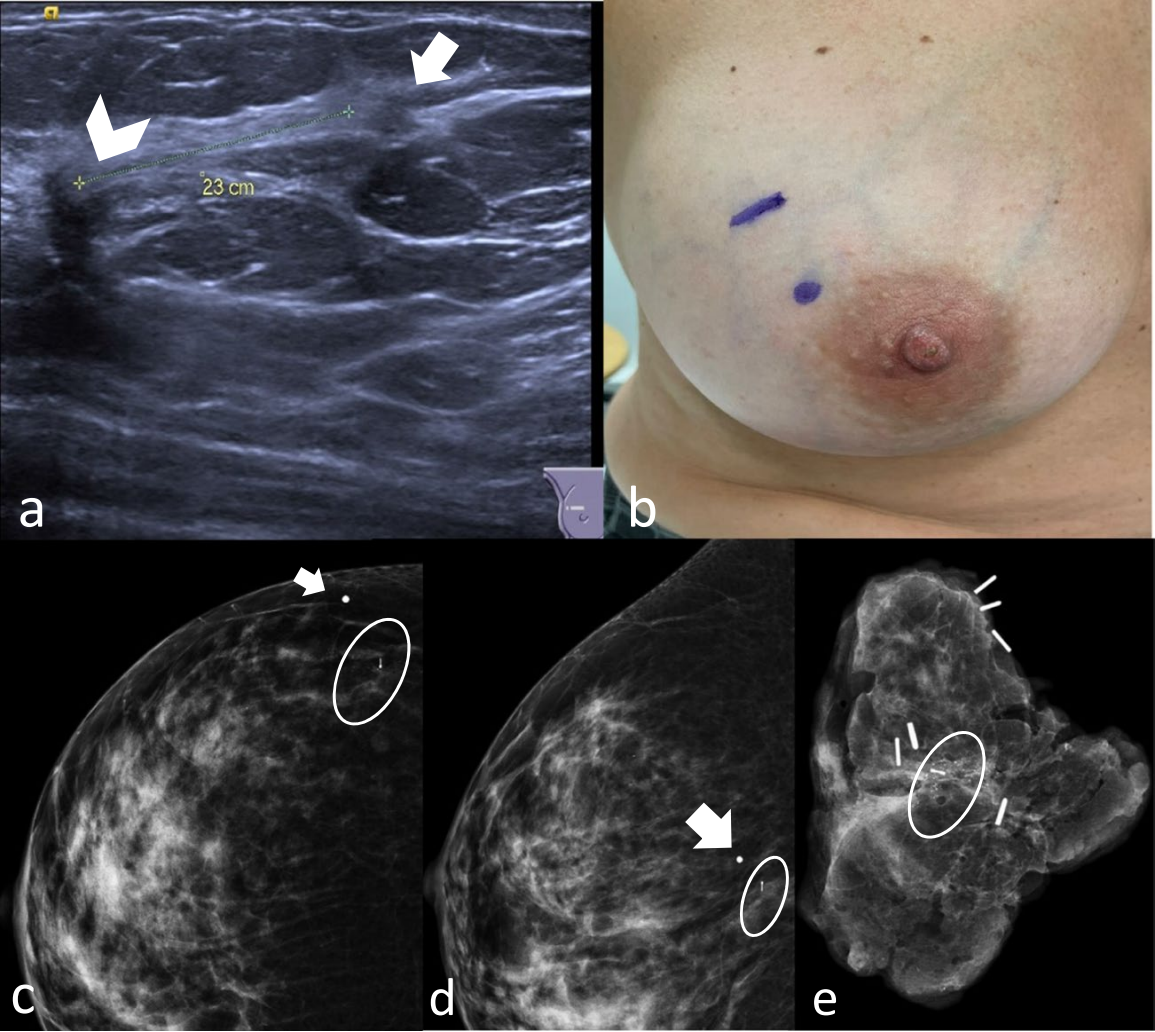
Ultrasound- and stereotactic-guided skin tattoo localizations. In **a**, two hypoechoic irregular masses (multifocal invasive ductal carcinoma) in the right upper-outer quadrant, respectively of 7 mm (arrowhead) and 4 mm (arrow), at a 23-mm interlesion distance. The dermographic skin markers of the tumor’s projection on the skin surface (**b**). Cranio-caudal (**c**) and mediolateral (**d**) views document the correct placement of the metallic marker (arrows in **c** and **d**) on the projection of microcalcifications and clip (circles) on the skin surface. Radiograph of specimen demonstrates the correct removal of the microcalcifications and the clip (**e**, circle)

Skin tattoo is conducted using mammography or ultrasound, the same day of the surgery or the day before. Ultrasound guidance is reserved to lesions detectable by the ultrasound examination, performed by positioning the probe on the target, without applying pressure, with the patient in supine position and the ipsilateral arm abducted, in the surgical position (Fig. 2). The depth of the lesion from the skin was measured with ultrasound.

Stereotactic guidance is performed in case of microcalcifications, architectural distortion, and clip deployed at the end of biopsy, not detectable with the ultrasound. A metallic marker is placed on the projection of the tumor on the skin surface based on previous mammography (Fig. 2). In case of lesions with significant size or widespread microcalcifications, more than one marker is used to delimit the extension of the area. Two-view mammogram (mediolateral oblique and cranio-caudal views) is acquired to confirm the correct position of the metallic marker before performing the skin tattoo.

### Surgical specimen

**Fig. 3:**
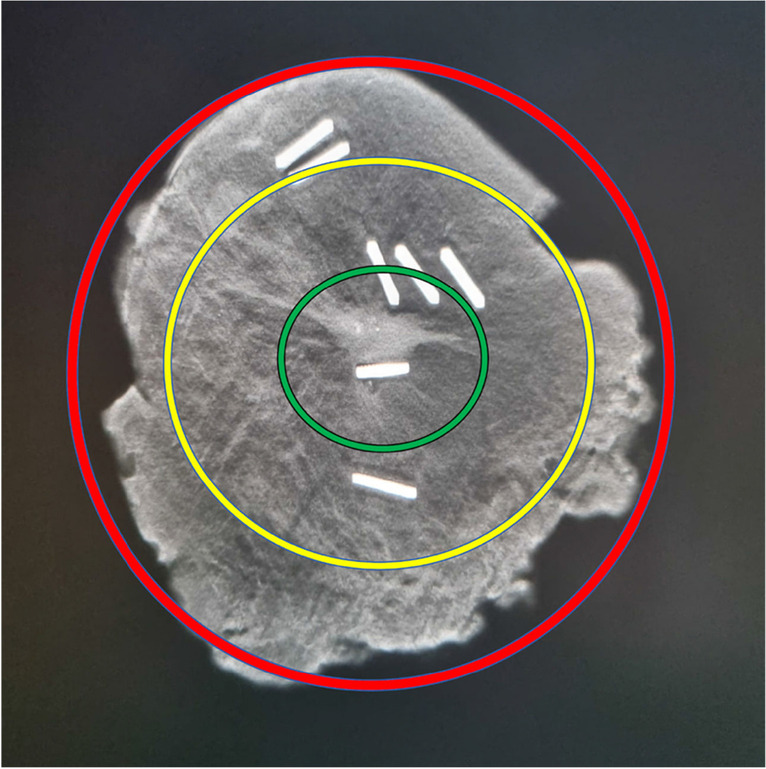
Surgical specimen divided in three concentric circles: circle 1 (green), circle 2 (yellow), and circle 3 (red)

Surgical specimen radiography in two orthogonal planes (cranio-caudal and mediolateral oblique views) was acquired after the excision to confirm the accuracy of surgical procedure in terms of presence of the lesion and clip or magnetic seed when present. For this purpose, to make a more standardized assessment of the lesion site in the specimen radiography, the surgical specimen was divided into three concentric circles (Fig. 3). The volume of the excised breast specimen was calculated as a geoid [13]. The specimen was transferred to the pathology department, first for macroscopic intraoperative assessment of the surgical margin status in terms of “standard” negative margins (≥ 2 mm) and then for the permanent section evaluation of margin status (“no ink on tumor”) [2].

### Statistical analysis

All data and statistical analysis were carried out using SPSS (version 26.0, SPSS Inc.). Continuous variables were presented as means ± standard deviation (medians and interquartile ranges), while the categorical variables were summarized as numbers and percentages. Fisher’s exact test was used to compare categorical variables and ANOVA test for continuous variables. A *p* < 0.05 was considered statistically significant. We compared demographics, breast size, site and size of the lesions, and radiological and surgical characteristics between the two groups.

## Results

Seventy-seven patients were included in the study. Median age was 58.06 ± 12.53 (57; 48.5–68) years. Anatomic, epidemiological, and radiological characteristics of enrolled patients were collected to ensure similar patient populations (Table S1). Thirty-seven magnetic seeds were placed in 36 patients (48.6%), two seeds were located in the same breast at a 2 cm distance. Forty skin tattoos were performed in 40 patients (51.4%).

No statistically significant difference was found for the histological characteristics between the two groups of patients (*p* = 0.976; Table S1). Moreover, no differences were found for the ultrasound characteristics in the two groups (*p* = 0.280; Table S1).

The site of the lesions was similar in the study population (*p* = 0.536), with a prevalence of the upper-outer quadrant.

Thirty-three magnetic seeds (89.2%) were placed under ultrasound guidance and 4 (10.8%) under stereotactic guidance. Twenty-three skin tattoos (57.5%) were performed under stereotactic guidance, and 17 (42.5%) under ultrasound guidance (Table 1). The seeds were placed correctly at the two-view mammogram acquired after the insertion in 97.6% (36/37) of cases. No seed migration was reported at the mammogram acquired after the marker insertion.

**Table 1:** Localization data

	Cohort	Skin tattoo	Magseed
	*N* = 77	*N* = 40 (51.9%)	*N* = 37 (48.1%)
US-guided placement	56 (72.7%)	17 (42.5%)	33 (89.2%)
Stereotactic placement	21 (27.3%)	23 (57.5%)	4 (10.8%)

Time (in minutes, min) spent for the localization was quite the same in the two groups (11.7 min for skin tattoo and 13.2 min for Magseed, *p* = 0.236; Table S2). The time (in days) between localization procedure and surgery was longer in the Magseed group (4.08 ± 15.8 days and 0.40 ± 0.55 days for seed and skin tattoo, respectively; Table S2).

The time taken to detect the lesions after the skin incision was significantly different in the two groups (*p* = 0.008), longer in Magseed cases (≥ 70 min in 12 cases; Table S2). The identification and selective excision of the lesions marked were successful in 100% of cases.

Considering the volume of surgical specimen, it was smaller in the magnetic seed group (33.68 ± 19.39 cm^3^ vs 61.63 ± 81.26 cm^3^, *p* = 0.046; Table S2). Regarding the breast volume and surgical specimen volume ratio, a statistically significant difference was observed in the seed group (0.98 ± 0.56 for Magseed and 2.56 ± 3.05 for skin tattoo, *p* = 0.003), with less breast parenchyma removed. Stratifying the data according to lesion dimensions, a smaller volume of breast parenchyma was removed in the magnetic seed group, especially in lesions smaller than 8 mm (0.93 ± 0.47 for Magseed and 2.4 ± 2.1 for skin tattoo, *p* = 0.019; Table S2).

The accuracy of the resection assessed with surgical specimen was excellent in both groups, with 51.9% of the total lesions sited in circle 1, of whom 52.5% localized with skin tattoo, and 51.4% with Magseed (Table S2).

The percentage of positive margins (≥ 2 mm) found at the macroscopic intraoperative assessment were the same for the two groups (29.7% and 30% for skin tattoo and Magseed respectively, *p* = 1.000; Table S2). The percentage of “ink on tumor” was slightly higher in localizations with skin tattoo (12.5%) compared to seed localizations (8.1%), without statistically significant differences (*p* = 0.401; Table S2).

Intraoperative widening was performed in 40% of cases localized with skin tattoo and 29.7% with magnetic seed (Table S2). The re-excision rate found in the cohort was 0%.

## Discussion

In our Breast Unit, about 1400 breast cancer patients are treated per year and the most used localization method of non-palpable breast lesions is skin tattoo. In our experience, this method is valid and effective, with a low rate of re-excision [14].

To the author’s knowledge, this is the first study comparing magnetic seed localization with skin tattoo.

The magnetic seed was successfully located in almost 100% of the cases (97.6%). The time demanded in the two localization procedures was almost the same (Table S2), though we expected a longer time for the seed placement, probably due to the familiarity of the radiologists of the breast unit with interventional procedures.

The possibility to place the Magseed days before surgery, contrary to the skin tattoo (4.08 ± 15.8 days for the seed group and 0.40 ± 0.55 days for skin tattoo; Table S2), helps in scheduling the workflow, avoiding localization on the same day of surgery.

A correlation between breast size and lesion depth from the skin surface was observed; indeed, the majority of magnetic seeds were placed in medium breasts (B-cup, 62.2%) with deeper lesions (depth was 15.7 mm and 12.2 mm for magnetic seed and skin tattoo respectively), not deeper than 40 mm from the skin surface, according to the literature [15, 16]. Moreover, Magseed was used more frequently in patients with lesions smaller than 5 mm (18.9%).

The less breast parenchyma removed in the Magseed group (breast/surgical specimen volume ratio 0.98 ± 0.56 vs 2.56 ± 3.05, *p* = 0.003; Table S2), especially in case of lesions smaller than 8 mm, revealed that the seed compared with skin tattoo allows to achieve a better esthetic result, without reducing the accuracy. Indeed, the accuracy of the two localization techniques in terms of positive margin (*p* = 1.000) and “no ink on tumor” (*p* = 0.401) was the same. The intraoperative widening performed did not differ in the two groups (*p* = 0.474), and it was in line with the literature [2].

The re-excision rate reported was 0% for both groups, lower than the generally accepted percentage of 20–25% [17], due to the administration of loco-regional radiation therapy after surgery according ASCO [18].

The removal of magnetic seed required longer surgical time (*p* = 0.008), probably due to the surgeon’s learning curve with this new technique introduced in our center in the last years, compared with skin tattoo, used for at least 10 years. This result was not in line with the literature that registered the same duration of surgical cases with minimal learning curve [19, 20]. In future larger studies, we will investigate this data to assess any performance changes.

With both methods, 100% of the index lesions were completely removed and found in the surgical specimen, with 51.9% of the total lesions in circle 1 (52.5% localized with skin tattoo and 51.4% with Magseed).

Our study has several limitations. Firstly, it was a single-center retrospective study without a randomization. Secondly, data on cost-effectiveness were not evaluated. Moreover, due to the retrospective nature of the study, no data were collected on patient satisfaction of the different techniques. Finally, the sample size was small. Prospective and larger studies are needed to confirm our results.

## Conclusions

Magnetic seed is a non-radioactive localization technique, feasible to place, recommended in case of non-palpable breast lesions, saving the breast parenchyma removed compared with skin tattoo, without reducing accuracy.

### Supplementary Information

Below is the link to the electronic supplementary material.

Supplementary file1 (PDF 123 KB)

## Abbreviations

ASCO: American Society of Clinical Oncology
FDA: Food and Drug Administration
MDT: Multidisciplinary team
ROLL: Radio-guided occult localization
RSL: Radioactive seed localization
WGL: Wire guide localization

## Declarations

### Guarantor

The scientific guarantor of this publication is Paolo Belli.

### Statistics and biometry

One of the authors has significant statistical expertise.

### Study subjects or cohorts overlap

No study subject or cohorts overlap to report.

### Methodology

• retrospective

• diagnostic study

• performed at one institution
