# Supplementary material for: Magnetic seed versus skin tattoo localization of non-palpable breast lesions: a single institution cohort study
Source: Eur Radiol. 2023 Aug 1;34(1):149–54. doi: 10.1007/s00330-023-10008-4 (PMC10791905; doi:10.1007/s00330-023-10008-4)
Supplement: Supplementary file 1 — Supplementary file1 (PDF 123 KB) [file 330_2023_10008_MOESM1_ESM.pdf]

Magnetic Seed versus skin tattoo localization of non-palpable breast lesions: a single institution cohort study

ELECTRONIC SUPPLEMENTARY MATERIAL

Table S1

Demographic and clinical data. DCIS: ductal carcinoma in situ; IDC: invasive ductal carcinoma; LCIS: lobular cancer in situ; LIC: lobular invasive cancer; B3 lesions; ICM: invasive mucinous carcinoma. UOQ: upper-outer quadrant; UIQ: upper-inner quadrant; LIQ: lower-inner quadrant; LOQ: lower-outer quadrant; RA: retroareolar. Millimeters (mm).

	Cohort	Skin-tattoo	Magseed	p-value
	N=77	N=40 (51,9%)	N=37 (48,1%)	
**Anatomic and epidemiological characteristics**				
Age, years	58.06 ± 12.53	57.88 ± 13.51	58.27 ± 11.56	p=0.891
	N (%)	N (%)	N (%)	
Breast size				
- Small (A-cup)	26 (33.8%)	19 (47.5%)	7 (18.9%)	
- Medium (B-cup)	39 (50.6%)	16 (40.0%)	23 (62.2%)	
- Large (C-cup)	12 (15.6%)	5 (12.5%)	7 (18.9%)	
Lesion site				p=0.536
- UOQ	53 (68.8%)	26 (65.0%)	27 (73.0%)	
- UIQ	8 (10.4%)	5 (12.5%)	3 (8.1%)	
- LIQ	11 (14.3%)	5 (12.5%)	6 (16.2%)	
- LOQ	2 (2.6%)	1 (2.5%)	1 (2.7%)	
- RA	3 (3.9%)	3 (7.5%)	0 (0%)	

Depth of lesion (mm)<br>- ≤ 9<br>- 10 – 20<br>- ≥ 21	8 (10.4%)<br>64 (83.1%)<br>5 (6.5%)	8 (20.0%)<br>30 (75.0%)<br>2 (5.0%)	0 (0%)<br>34 (91.9%)<br>3 (8.1%)	
**Imaging characteristics**				
Mammography findings<br>- Masses<br>- Microcalcifications<br>- Architectural distortion<br>- Negative<br>- Clip	31 (40.3%)<br>27 (35.1%)<br>10 (13.0%)<br>6 (7.8%)<br>3 (3.9%)	17 (42.5%)<br>19 (47.5%)<br>2 (5.0%)<br>2 (5.0%)<br>0 (0%)	14 (37.8%)<br>8 (21.6%)<br>8 (21.6%)<br>4 (10.8%)<br>3 (8.1%)	p=0.014
Ultrasound findings<br>- Masses<br>- Heterogeneous area<br>- Negative	56 (72.7%)<br>18 (23.4%)<br>3 (3.9%)	28 (70.0%)<br>11 (27.5%)<br>1 (2,5%)	28 (75.7%)<br>7 (18.9%)<br>2 (5.4%)	p=0.280
Histological diagnosis<br>- DCIS<br>- IDC<br>- LCIS<br>- LIC	5 (6.6%)<br>50 (65.8%)<br>1 (1.3%)<br>8 (10.5%)	2 (5.1%)<br>26 (66.7%)<br>1 (2.6%)<br>4 (10.3%)	3 (8.1%)<br>24 (64.9%)<br>0 (0%)<br>4 (10.8%)	p=0.976

- B3<br>- ICM	11 (14.5%)<br>1 (1.3%)	6 (15.4%)<br>0 (0%)	5 (13.5%)<br>1 (2.7%)	

Table S2

Surgical outcomes. Millimeters (mm); minutes (min); days (dy)

Characteristics	Cohort	Skin tattoo	Magseed	p-value
	N=77	N=40 (51.9%)	N=37 (48.1%)	
Lesion dimension (mm)	12.62 ± 10.13	16.23 ± 12.51	8.73 ± 4.16	
	N (%)	N (%)	N (%)	
Lesion dimension (mm)				
- ≤ 5	9 (11.7%)	2 (5.0%)	7 (18.9%)	
- 6 – 15	45 (58.4%)	21 (52.5%)	24 (64.9%)	
- ≥ 16	23 (29.9%)	17 (42.5%)	6 (16.2%)	
Time of localization (min)	24.9 (100%)	11.7 (46.9%)	13.2 (53.1%)	p=0.236
Time between localization and surgery (dy)	2.17 ± 11.06	0.40 ± 0.55	4.08 ± 15.84	
Time from skin incision to surgical excision (min)				
- ≤ 45	32 (41.6%)	19 (47.5%)	13 (35.1%)	p=0.008
- 46 – 69	31 (40.3%)	19 (47.5%)	12 (32.4%)	
- ≥ 70	14 (18.2%)	2 (5.0%)	12 (32.4%)	
Type of surgery				
- Excisional biopsy	3 (3.9%)	0 (0%)	3 (8.1%)	p=0.053
- Lumpectomy	72 (93.5%)	38 (95.0%)	34 (91.9%)	

- Mastectomy	2 (2.6%)	2 (5.0%)	0 (0%)	
Lesion site in specimen radiogram<br>- Circle 1<br>- Circle 2<br>- Circle 3	40 (51.9%)<br>26 (33.8%)<br>11 (14.3%)	21 (52.5%)<br>13 (35.1%)<br>6 (15.0%)	19 (51.4%)<br>13 (35.1%)<br>5 (13.5%)	p=1.000
Intraoperative surgical margins (≥2mm)<br>- Negative<br>- Positive	54 (70.1%)<br>23 (29.9%)	28 (70.0%)<br>12 (30.0%)	26 (70.3%)<br>11 (29.7%)	p=1.000
Intraoperative widening<br>- No<br>- Yes	50 (64.9%)<br>27 (35.1%)	24 (60.0%)<br>16 (40.0%)	26 (70.3%)<br>11 (29.7%)	p=0.474
'Ink on tumor'	8 (10.4%)	5 (12.5%)	3 (8.1%)	p= 0.401
Volume of surgical specimen (cm^3)	48.29 ± 61.23	61.63 ± 81.26	33.68 ± 19.39	p=0.046
Breast/surgical specimen volume ratio (cm^3)	1.80 ± 2.36	2.56 ± 3.05	0.98 ± 0.56	p=0.003
Cancer lesion < 8 mm				
Breast/surgical specimen volume ratio (cm^3)	1.80 ± 2.36	2.39 ± 2.07	0.93 ± 0.47	p=0.019
